# Sexual activity increases the number of newborn cells in the accessory olfactory bulb of male rats

**DOI:** 10.3389/fnana.2012.00025

**Published:** 2012-07-06

**Authors:** Wendy Portillo, Nancy Unda, Francisco J. Camacho, María Sánchez, Rebeca Corona, Dulce Ma. Arzate, Néstor F. Díaz, Raúl G. Paredes

**Affiliations:** ^1^Instituto de Neurobiología, Universidad Nacional Autónoma de MéxicoQuerétaro, México; ^2^Instituto Nacional de PerinatologíaD.F., México

**Keywords:** sexual behavior, neurogenesis, accessory olfactory bulb and main olfactory bulb

## Abstract

In rodents, sexual behavior depends on the adequate detection of sexually relevant stimuli. The olfactory bulb (OB) is a region of the adult mammalian brain undergoing constant cell renewal by continuous integration of new granular and periglomerular neurons in the accessory (AOB) and main (MOB) olfactory bulbs. The proliferation, migration, survival, maturation, and integration of these new cells to the OB depend on the stimulus that the subjects received. We have previously shown that 15 days after females control (paced) the sexual interaction an increase in the number of cells is observed in the AOB. No changes are observed in the number of cells when females are not allowed to control the sexual interaction. In the present study we investigated if in male rats sexual behavior increases the number of new cells in the OB. Male rats were divided in five groups: (1) males that did not receive any sexual stimulation, (2) males that were exposed to female odors, (3) males that mated for 1 h and could not pace their sexual interaction, (4) males that paced their sexual interaction and ejaculated one time and (5) males that paced their sexual interaction and ejaculated three times. All males received three injections of the DNA synthesis marker bromodeoxyuridine at 1h intervals, starting 1 h before the beginning of the behavioral test. Fifteen days later, males were sacrificed and the brains were processed to identify new cells and to evaluate if they differentiated into neurons. The number of newborn cells increased in the granular cell layer (GrCL; also known as the internal cell layer) of the AOB in males that ejaculated one or three times controlling (paced) the rate of the sexual interaction. Some of these new cells were identified as neurons. In contrast, no significant differences were found in the mitral cell layer (also known as the external cell layer) and glomerular cell layer (GlCL) of the AOB. In addition, no significant differences were found between groups in the MOB in any of the layers analyzed. Our results indicate that sexual behavior in male rats increases neurogenesis in the GrCL of the AOB when they control the rate of the sexual interaction.

## Introduction

In rodents, male sexual behavior relies on the male's ability to identify a conspecific and to determine if she is sexually receptive. Sexually active females and males emit many kinds of stimuli that affect several sensory modalities to attract the opposite sex. Several studies have demonstrated that olfactory cues are crucial for the appropriate selection of a sexual partner in rodents (Curtis et al., 2001; Brennan and Kendrick, 2006; Baum and Kelliher, 2009).

The accessory and the main olfactory systems are functionally and anatomically interrelated. Thus, the main olfactory system can respond to odors that effectively activate the accessory olfactory system and neurons in the vomeronasal organ can be activated by volatile and non volatile odorants (Trinh and Storm, 2003; Xu et al., 2005; Levai et al., 2006; Spehr et al., 2006). Both systems respond sequentially to sexually relevant cues, responsible for social responses to chemical signals from conspecifics (Xu et al., 2005; Martel and Baum, 2007; Jakupovic et al., 2008; Slotnick et al., 2010). There are, in fact two sites of interaction between the main (MOB) and accessory (AOB) olfactory bulb (OB) namely, interstitial neurons of the bulbi (INBs) and the main accessory cells (MAC) (Larriva-Sahd, 2008).

In male rats, mating activates the granular (GrCL; also known as the internal) and mitral (MCL; also known as the external) cell layers of the AOB as evaluated by expression of the immediate early gene cFos (Kondo et al., 2003). Surgical removal of the vomeronasal organ decreases basal as well as mating induced cFos in the GrCL (Kondo et al., 2003). Sexually experienced male rats with lesions of the vomeronasal organ show an increase in the number of mounts and a longer intromission and ejaculation latency (Saito and Moltz, 1986; Kondo et al., 2003). Sexually naive male rats with lesions of the vomeronasal organ also show longer intromission and ejaculation latencies and higher number of intromissions (Saito and Moltz, 1986). On the other hand, in sexually experienced male rats, olfactory preference for sexually active females is impaired by lesions of the olfactory epithelium (Dhungel et al., 2011). These lesions decrease intromission frequency and increase ejaculation and mount latency (Dhungel et al., 2011). These studies clearly indicate that the olfactory systems play a crucial role in the modulation of male sexual behavior.

The OB is a region where new neurons are continuously added during adult life (Alvarez-Buylla and Garcia-Verdugo, 2002). The new cells originate in the subventricular zone, which contains neuronal stem cells that generate neuroblasts (Reynolds and Weiss, 1992; Lois et al., 1996). The neuroblasts travel along the rostral migratory stream toward the OB using tangential chain migration (Lois et al., 1996; Peretto et al., 1997). Once they reach the OB the immature cells detach from the migratory stream and migrate radially toward different layers of the MOB and AOB (Peretto et al., 2001; Belluzzi et al., 2003; Carleton et al., 2003; Curtis et al., 2007; Oboti et al., 2009). Approximately 95% of the new neurons differentiate into GABAergic granular interneurons (Lledo and Saghatelyan, 2005; Bagley et al., 2007). A minority become GABAergic periglomerular interneurons (Bagley et al., 2007; Batista-Brito et al., 2008; Whitman and Greer, 2009; Ming and Song, 2011). Petreanu and Alvarez-Buylla (2002) describe five stages in the differentiation of adult born granular cells in mice. In stage 1 (days 2–7) cells migrate to the rostral migratory stream, in stage 2 (days 5–9) the new cells initiate radial migration to the OB. In stage 3 (days 9–13) they reach their final position, in stage 4 (days 11–22) the new cells develop dendrite arbors, and finally in stage 5 (days 21–42) the new granular cells show a mature morphology (Petreanu and Alvarez-Buylla, 2002). Between 15 and 45 days after birth, approximately 50% of the newly generated granule cells die and the other half is integrated in the OB.

The survival of the new cells depends on the level of activity that they received (Petreanu and Alvarez-Buylla, 2002; Winner et al., 2002). For example, it has been demonstrated that in male mice the prolonged exposure (40 days) to an odor enriched environment increases the number of new cells in the glomerular cell layer (GlCL) of the MOB facilitating odor discrimination (Rochefort et al., 2002; Rochefort and Lledo, 2005). Sexually relevant odors also increase neurogenesis in the OB (Mak et al., 2007; Larsen et al., 2008; Oboti et al., 2009). Exposure of female mice for seven days to pheromones from a dominant, but not from a subordinate male, induced an increase in the number of new cells in the subventricular zone and dentate gyrus. This increase in the number of new cells had functional relevance because females exposed to soiled bedding from dominant males showed a clear preference for these males instead of subordinate males. This preference was eliminated if the females were treated with a mitotic inhibitor in order to prevent neurogenesis (Mak et al., 2007). The new cells that are integrated into the OB express cFos in response to estrous female odors and mating stimulation in male hamsters (Huang and Bittman, 2002). Taken together, these studies suggest that the new cells that reach the OBs can participate in the processing of socio-sexual relevant signals.

Sexual behavior also induces cell proliferation and neurogenesis. Males that mated once or several times show an increase in cell proliferation and neurogenesis in the dentate gyrus of the hippocampus. No changes were observed in those males only exposed to receptive females (Leuner et al., 2010). Our laboratory was the first to demonstrate that sexual stimulation in females increases the number of new cells in the OB. Female rats that paced the sexual interaction showed an increase in the number of new cells that reach the GrCL of the AOB 15 days after mating. No significant differences were found in the number of new cells in GlCL, or MCL of the AOB. Nor were any significant differences found in the cell layers of the MOB (Corona et al., 2011b). The aim of the present study was to evaluate if sexual behavior in male rats increases the number of new cells that reach the OB and if those new cells differentiate into neurons. We evaluated the number of new cells in the OB 15 days after sexual stimulation because at this time more new cells arrive to the layers of the OB and develop spines (Petreanu and Alvarez-Buylla, 2002; Winner et al., 2002).

For this purpose the following groups were included: (1) control males, that did not receive any sexual stimulation, (2) males exposed for 1 h to a sexually receptive female, (3) males that mated for 1 h in a setting where they were not able to pace the sexual interaction, (4) males that paced their sexual interaction with a receptive female until they ejaculated once and (5) males that paced their sexual interaction until they ejaculated three times. We predicted that those males that control the rate (pace) of the sexual interaction would have a higher number of new cells in the GrCL but not in the MCL or GlCL layer of the AOB. In addition, since no changes were observed in the MOB when females paced the sexual interaction we did not expect differences in any of the layers of the MOB. We also evaluated if some of the new cells differentiate into neurons.

## Methods

### Animals

Sexually naive male Wistar rats (250–350 g) were obtained from the local breeding colony at the Instituto de Neurobiología de la Universidad Nacional Autónoma de México. Subjects were maintained in a room with controlled temperature (25 °C) and humidity and under a reverse dark-light cycle (12–12 h). Standard laboratory rat chow and water were available ad libitum. Sexually experienced female Wistar rats (200–300 g) were used as stimulus. They were gonadectomized and brought into estrous by hormone treatment with 25 μg/rat of estradiol benzoate (Sigma) 48 h before and with 1mg/rat of progesterone (Aldrich) 4 h before the mating test (Gonzalez-Flores et al., 2004; Arzate et al., 2011; Corona et al., 2011a). In order to acquire sexual experience males mated in a condition where they were allow to pace the sexual interaction with sexually receptive females in three 30 min tests, one test per week. Only those males that ejaculated in each test were included in the experiment. All experiments were carried out in accordance with the “Reglamento de la Ley General de Salud en Materia de Investigación para la Salud” of the Mexican Health Ministry.

### Apparatus

The behavioral tests were performed in clear acrylic cages (40 × 60 × 40 cm), with fresh sawdust covering the cage floor.

### Experimental design

#### Groups

**Figure 1:**
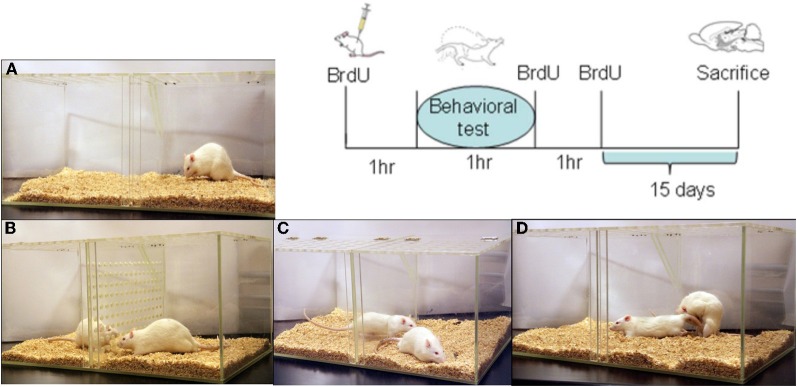
**Representation of the different groups (A–D) and sequence of BrdU injections for all subjects.** The control group **(A)** was placed in the mating cage for 1 h; in group **(B)** males were exposed without physical contact to a sexually receptive female; in group **(C)** the males mated but could not control the rate of the sexual interaction, while males in group **(D)** were allowed to pace the sexual interaction until 1 ejaculation or until 3 ejaculations. BrdU treatment (100 mg/kg per injection; 3 injections) was administered 1 h before the behavioral test (day 0), at the end of the test, and 1 h after the test. Subjects were sacrificed 15 days after the behavioral test.

Males were randomly divided into five groups (*N* = 7 each) as follows: group (1) males were placed for 1 h in a clean mating cage without sexual or olfactory stimulation (control, C); group (2) males were exposed for 1 h to female odors (EXP). They were placed in a mating cage divided in two equal compartments by an acrylic screen with 1 mm diameter holes. The experimental male was placed on one side of the cage and the sexually receptive female on the opposite side; in this way, males were able to smell, see and hear the stimulus female without physical contact; group (3) males mated for 1 h in a setting where they were not able to pace the sexual interaction (NP). In this group the mating cage was divided by a removal clear screen with a small hole at the bottom that allowed the female, but not the male, to move freely from one side of the cage to the other. In this condition the male is not able to pace the sexual interaction; group (4) males paced their sexual interaction with a receptive female until they ejaculated one time (1E); group (5) males paced their sexual interaction until they ejaculated three times (3E), see Figure 1. Each male always mated with the same female to avoid possible effects of female novelty on the number of new cells.

### Sexual behavior parameters

In the sexual behavioral tests, the following parameters were recorded: number of mounts (M), intromissions (I), and ejaculations (E); the latency to first M, I, and E; the post ejaculatory interval (PEI) and the inter-intromission interval (III, ejaculation latency/number of intromission before ejaculation).

### BrdU administration

On day 0 of the experiment, males were injected intraperitoneally three times with the DNA synthesis marker 5′-bromo-2-deoxyuridine (BrdU; Sigma, dissolved in saline solution). They were injected: (1) 1 h before the test, (2) immediately after the behavioral test and (3) 1 h after the test. This procedure allows labeling of the new cells synthesized around the time of the behavioral test. Each BrdU administration was 100 mg/kg which means that each male received a total of 300 mg/kg of the DNA synthesis marker (Figure 1).

Fifteen days after BrdU administration, all males were injected with a lethal dose of sodium pentobarbital (100 mg/kg). They were perfused with 0.1 M phosphate-buffered solution (PBS, pH 7.4) followed by 4% paraformaldehyde in 0.1 M PBS. The brains were removed from the skull and postfixed in 4% paraformaldehyde for 1 h before they were placed in 30% sucrose dissolved in 0.1 M PBS solution for cryoprotection. The two brain hemispheres were separated and the right half was sliced in the sagittal plane at 35 μm using a sledge microtome. Brain slices were divided in two series; one was processed for immunohistochemistry and the other for immunofluorescence.

### Immunochemistry

**Figure 2:**
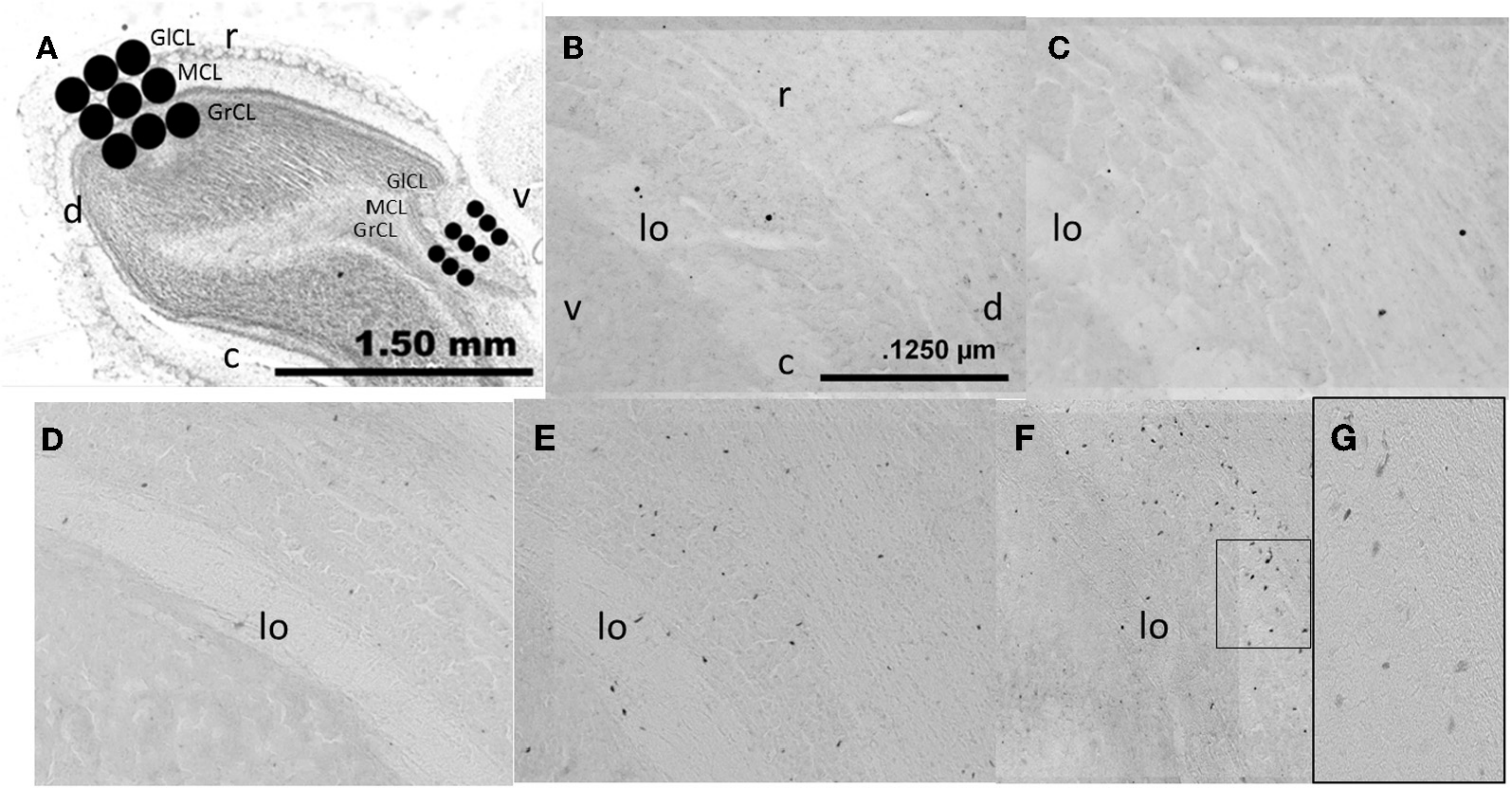
**Photomicrographs showing the layers analyzed in the sagittal (A) plane in the AOB and MOB, glomerular cell layer (GlCL), mitral cell layer (MCL), and granular cell layer (GrCL).** Representative photomicrographs (10×) showing BrdU positive cells in the GrCL of the AOB in control **(B)**, exposed **(C)**, non-paced **(D)**, 1 ejaculation **(E)** and 3 ejaculation **(F)** groups. A 40× magnification of the 3 ejaculation group is shown in **(G)**. lo, lateral olfactory tract; r, rostral; c, caudal; d, dorsal and v, ventral.

Brain slices were processed as previously reported (Corona et al., 2011b). Briefly, free-floating sections were incubated for at least 16 h at 4°C in primary antibody mouse monoclonal anti-BrdU (1:2,000; BD Bioscience). Later, primary antibody was removed by rinsing and the tissue was incubated for 2 h at room temperature with the secondary antibody biotinylated goat anti-mouse IgG (1:500; Vector laboratories). Brain sections were then rinsed and incubated in Avidin Biotin Complex (AB elite kit; Vector Laboratories) for 90 min at room temperature. Brain sections were rinsed and revealed with the chromogen solution nickel chloride-3,3′-diaminobenzidine (DAB; Vector Laboratories) and H_2_O_2_. Finally, the reactions were stopped by washing the slices in buffer solution. The brain slices were mounted onto gelatin-coated slides and cover slipped using permount. For each animal, three sections were analyzed, and the average was used for statistical analysis. The OB was reconstructed using microphotographs (10×) taken in a light microscope (Olympus BX60) connected to a motorized slide (Prior ProScan) and analyzed by Image Pro software. Sagittal brain sections were selected at the level of the AOB and MOB; we analyzed the GrCL, MCL, and GlCL layers (Figure 2A). The area of interest was delimited on the AOB by three circles of 200 μm diameter and in the MOB by 400 μm diameter circles placed in the rostral medial region. We chose these locations because we and other groups have evaluate neurogenesis process in this region (Bagley et al., 2007; Whitman and Greer, 2009; Corona et al., 2011b), and cells in these areas are activated during the processing of relevant odors (Keller et al., 2006). Data were expressed as the number of new cells per area in order to compare with our previous studies in female rats and in accordance with reports that evaluate the neurogenesis process in the OB (Alonso et al., 2006; Mouret et al., 2008; Honda et al., 2009; Mouret et al., 2009; Corona et al., 2011b).

### Immunofluorescence

In order to evaluate if the new cells (BrdU positive) differentiate into neuron lineage we measured immunofluorescence of neuronal nuclei. For this analysis we processed brains only from C, 1E, and 3E groups, because 1E and 3E groups showed an increased in the number of new cells in comparison to control males. As we already described (Corona et al., 2011b) sagittal brain sections were incubated for two nights at 4°C with the primary antibodies, rat monoclonal anti-BrdU (1:800; AbD serotec) and mouse monoclonal Neuronal Nuclei antibody (NeuN) to label mature neurons (1:250; CHEMICON). Later the samples were incubated with the secondary antibodies, anti-rat IgG Alexa Fluor 488 (1:1000; Invitrogen) and anti-mouse IgG Alexa Fluor 568 (1:1,000; Invitrogen), respectively. At the end of the procedure the brain sections were mounted and cover slipped using Aqua Poly/Mount (Polysciences, Inc.). At least three sections of three animals in each group were evaluated and the average of the sections per male were analyzed. Olfactory bulb reconstruction was made at 20× using a fluorescence lamp. Image-Pro software was used to merge photographs of the BrdU and NeuN positive cells. In order to verify the double labeled cells, the images were acquired in a LSM 510 Meta confocal microscope (Carl Zeiss, Germany) using a plan-neofluar 20× objective (N.A. 0.50, Zeiss), to detect Alexa 488 with Argon Laser in 488 nm wavelength and Alexa 568 was detected with DPSS Laser 561 nm wavelength fluorescence in a sequential fashion. To establish co-expression of the markers used, merged images were generated (Figure 4). The examiner was unaware of the groups of the slices.

## Statistics

Data from sexual behavior were not normally distributed and therefore were analyzed by a Kruskal-Wallis test followed by Student Newman-Keuls test. Data from the number of BrdU immunoreactive cells and number of BrdU/NeuN immunofluorescence cells were normally distributed and therefore analyzed by One-Way analysis of variance (ANOVA) for each layer. In case of significant effects *post hoc* comparisons were performed with Fisher's least significant difference test.

## Results

### Sexual behavior

During the three training tests to obtain sexual experience no significant differences between groups were observed in any of the parameters analyzed (data not shown). In the sexual behavior test performed when BrdU was injected we found a significant difference in the number of intromissions (K-W test: χ^2^ = 6.75, *P* = 0.034). The *Post hoc* test revealed that the 1E and 3E groups displayed a higher number of intromissions than the NP group. Significant differences were also found in III (K-W test: χ^2^ = 7.22, *P* = 0.027), the NP group had a longer III than 1E and 3E groups. Although the 3E and 1E groups had a higher number of M that NP males, this difference was not statistically different (K-W test: χ^2^ = 5.8, *P* = 0.054). As well, in the other parameters no significant differences were found: latency to the first M (K-W test: χ^2^ = 0.5, *P* = 0.79), I (K-W test: χ^2^ = 0.9, *P* = 0.65), E (K-W test: χ^2^ = 0.36, *P* = 0.83), and PEI (K-W test: χ^2^ = 4.4, *P* = 0.11) Table 1.

**Table 1:** **Sexual behavior parameters in the different groups; no pacing (NP), one ejaculation (1E), and three ejaculations (3E)**.

	**NP**	**1E**	**3E**
**Number**						
Mounts	6 ± 1	14.3 ± 3.2	8.1 ± 1.3
Intromissions	11 ± 1.2	20.4 ± 2.3^*^	18.7 ± 3^*^
Ejaculations	3.4 ± 0.2	1	3
**Latencies (sec)**						
Mounts	94.3 ± 25.3	183.1 ± 82.6	122.1 ± 40
Intromissions	103.6 ± 26.7	223.28 ± 95.9	154.1 ± 44.3
Ejaculations	937.9 ± 85.8	905.6 ± 189.2	903.9 ± 186
IPE	417.9 ± 36.6	356 ± 13.2	416.7 ± 21.3
III (sec)	94.7 ± 19.3	45.4 ± 9.3^*^	49.5 ± 8.1^*^

Data are expressed as mean ± SEM. N = 7 per group.

* Different from NP. P < 0.05.

### BrdU positive cells

#### AOB

Our data showed significant differences between groups in the number of new cells in the GrCL [*F*_(4, 34)_ = 11.64, *P* < 0.001]. *Post hoc* tests revealed an increase in the number of new cells in the 3E group compared to all other groups. We also found significantly more new cells in the 1E group than in groups C and NP.

**Figure 3:**
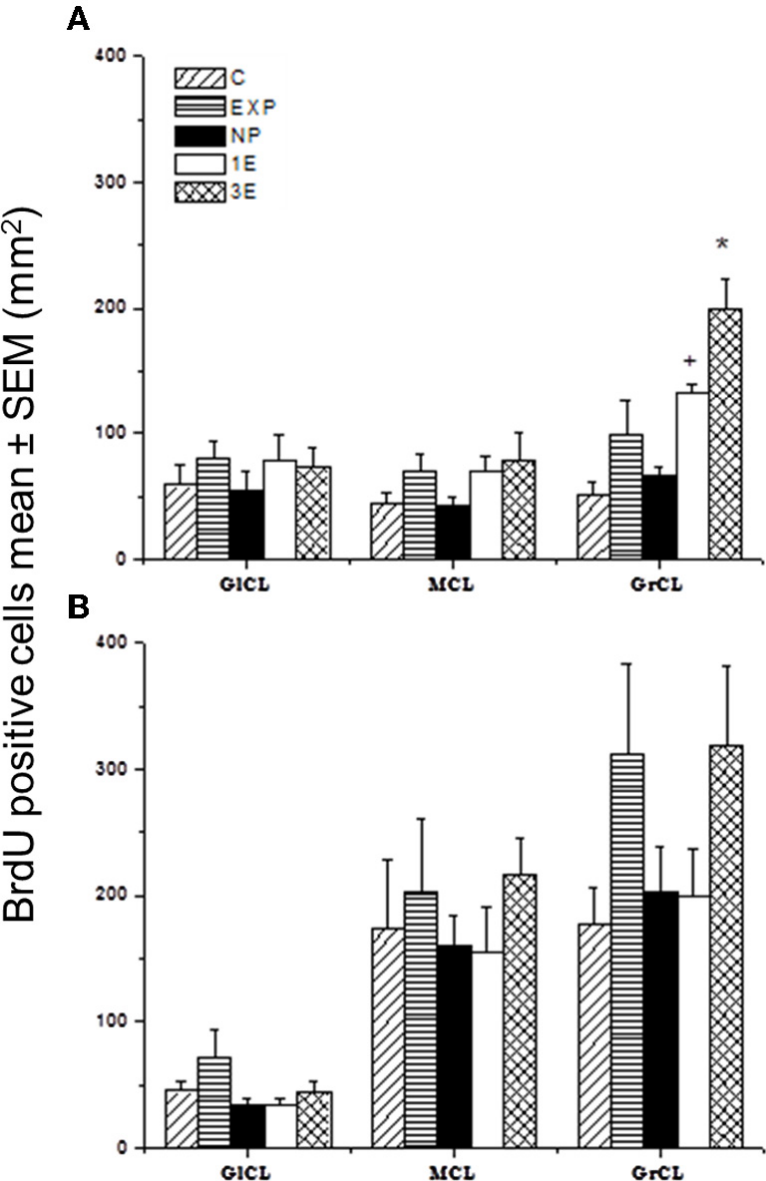
**Number of BrdU positive cells in the sagittal plane in the accessory olfactory bulb (AOB; A) and main olfactory bulb (MOB; B) in control (C), exposed (EXP), non-paced (NP), 1 ejaculation (1E), and 3 ejaculation (3E) groups.** The areas analyzed were the glomerular (GlCL), mitral (MCL) and granular cell layer (GrCL). Data are expressed as mean ± SEM. *N* = 7. ^*^Different from all other groups in the same layer. *P* < 0.05. +Different from C and NP in the same layer. *P* < 0.05.

No significant differences between groups were found in the number of new cells in the MCL [*F*_(4, 23)_ = 0.52, *P* = 0.72], nor did we find any significant differences in the GlCL [*F*_(4, 34)_ = 0.51, *P* = 0.74] (Figures 2B–G and 3A).

#### MOB

No significant differences were found between groups in the number of new cells in the GlCL [*F*_(4, 34)_ = 1.85, *P* = 0.15]; MCL [*F*_(4, 34)_ = 0.39, *P* = 0.82] or GrCL [*F*_(4, 34)_ = 1.49, *P* = 0.23] (Figure 3B).

We also evaluated the number of new cells in the coronal plane in the AOB and MOB to determinate if the results vary depending on the type of section. In the AOB, identical results were found, that is a significant greater number of BrdU positive cells in the GrCL in the 3E group compared to all other groups. A significant increase in the 1E group as compared to the C, EXP, and NP groups was also observed. No significant differences were found in the GlCL and ML (data not shown). In the MOB, as well as the sagittal sections, no significant differences were found in the coronal GlCL and MCL. However in the coronal GrCL we found that the 3E group integrates more cells than the 1E and control group (data not shown). This difference could be a spurious effect since the difference is marginal (*P* = 0.04) and has not been observed in females. Thus, in general the results did not depend on the type of section.

#### BrdU/NeuN positive cells

**Figure 4:**
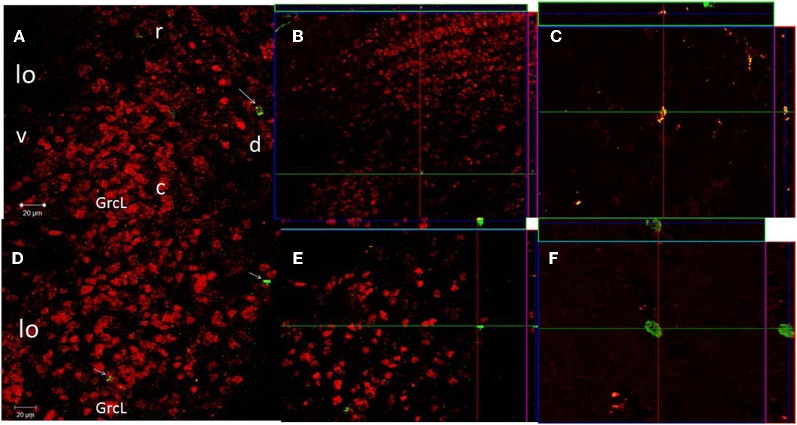
**Confocal images of cells in the GrCL of the AOB double-labeled with NeuN (red) and BrdU (green), taken at 20× magnification.** Projection and orthogonal plane in control **(A–C)**, and 3E **(D–F)** groups. The images were rotated in orthogonal planes to verify double labeling throughout its extent **(B,E**). Photomicrographs C and F were taken at 63× magnification. r, rostral; c, caudal; d, dorsal and v, ventral.

We quantified the number of new cells that expressed the marker of neuronal differentiation (BrdU/NeuN positives) in the GrCL in the C, 1E, and 3E groups because only between them we found significant differences in the number of new cells. Our data showed significant differences in the number of BrdU/NeuN positive cells [*F*_(2, 8)_ = 9.0, *P* = 0.02]. The *post hoc* test showed that the 3E group had more BrdU/NeuN positive cells per mm^2^ (BrdU positive cells 155.7 ± 20, BrdU/NeuN positive cells 69 ± 8, 44%) than the C (BrdU positive cells 51.3 ± 30.7, BrdU/NeuN positive cells 26.5 ± 9.2, 52%) and 1E groups (BrdU positive cells 106.2 ± 22, BrdU/NeuN positive cells 33.6 ± 4.7, 32%). In order to verify the colocalization of the double-labeled BrdU/NeuN cells we obtained 20× photomicrographs using a confocal microscope (Zeiss LSM 510) in the GrCL of the AOB, two sagittal slices from three randomly chosen subjects from the C, 1E and 3E groups were analyzed (Figure 4).

## Discussion

As expected, our data indicate that those males that ejaculated in a condition where they controlled the rate of sexual stimulation (paced mating) have a higher number of new cells in the GrCL of the AOB, regardless whether they ejaculated one or three times. We also showed that around 40% of these new cells differentiate into neurons within 15 days after mating and BrdU administration. Our results indicate that the increase in the number of new cells in the GrCL of the AOB depends on the ability to pace the sexual interaction more than on the number of ejaculations itself. The group of males not allowed to pace the sexual interaction ejaculated a mean of 3.4 times during the 1 h test (NP group) but did not show an increase in the number of new cells. Thus, paced genital stimulation appears to be fundamental to potentiate neurogenesis in the GrCL of the AOB in the adult male rat. The genital stimulation that the male receives during paced and non-paced mating is quantitatively and qualitatively different. Males that mate in a setting where they control (pace) the sexual interaction display a higher number of intromissions, longer intromission duration and a shorter inter-intromission interval in comparison to males not able to control the sexual interaction. Thus penile stimulation during intromissions is higher in those males that pace the sexual interaction (Erskine, 1989). The results of the present study further support this contention: males that paced the sexual interaction (1E and 3E) displayed a higher number of intromissions and had a shorter inter-intromission interval than the group unable to pace the sexual interaction (NP).

Another difference between paced and non-paced mating is the rewarding value of the sexual interaction. Sexual stimulation induces a reward state only if subjects, males or females, pace the sexual interaction (Martinez and Paredes, 2001; Camacho et al., 2009). This reward state is mediated by opioids since administration of the opioid antagonist naloxone, blocks the rewarding effects induced by sexual behavior in both males and females (Agmo and Gomez, 1993; Paredes and Martinez, 2001). Based on these and other observations it has been postulated that opioids are release during sexual behavior thereby reducing the aversive consequences of repeated sexual stimulation and enabling the eventual development of a reward or positive affective state (Agmo and Gomez, 1993; Paredes, 2009, 2010). Interestingly, opioids are also involved in the proliferation and survival of the new cells in the subventricular zone (Sargeant et al., 2008). Morphine treatment increased the number of new cells [detected by (3H) thymidine up take] in the subventricular zone of adult male rats (Messing et al., 1979; Miller et al., 1982). Furthermore, activation of the δ opioid receptor induced neuronal differentiation of brain stem cells, and the blockade of this opioid receptor induced astrogliosis (Narita et al., 2006). Therefore, endogenous opioid release during paced mating could be involved in the higher number of new neurons observed in the AOB.

Similar arguments could be presented for other neuromodulators. For example, oxytocin is also involved in the reward aspects of sexual interaction in females. Moreover, oxytocin release increases in the hypothalamic paraventricular nucleus of those females that pace the sexual interaction (Nyuyki et al., 2011). Additionally, oxytocin increases proliferation and adult neurogenesis in the ventral dentate gyrus of the hippocampus (Leuner et al., 2012). Further studies need to directly assess if blockade of either opioids or oxytocin during sexual behavior interferes with the increase of new neurons in the AOB.

It has been demonstrated that sexual stimulation activates neurons in the AOB. For example, Binns and Brennan demonstrated that mating in female mice induces a significant change in baseline activity in local field potential (LFP) in the granular cells of the AOB, resulting in significantly higher baseline LFP power in the frequency bands related to sensory perception and long-range interaction between brain regions (Binns and Brennan, 2005). Vaginal stimulation in females during proestrous-estrous also induces an increase in the firing rate in cells of the mitral cell layer of the MOB (Guevara-Guzman et al., 1997). In the same study, an increase in the number of cells that express cFos was reported in the GrCL of the AOB and in the GlCL and external plexiform layers of the MOB in proestrous-estrous females after vaginal stimulation (Guevara-Guzman et al., 1997).

Changes in olfactory function after exposure to relevant chemosensory cues and sexual stimulation are also observed in males. Subjects exposed to soiled estrous bedding showed an increase in the number of granular cells that express the immediate early gene, activity-regulated cytoskeleton-associated protein (ARC) in the MOB. Mating caused an even greater increase in the number of ARC-expressing granular cells in the MOB and in the AOB (Matsuoka et al., 2002). Moreover, in male rats genital stimulation increased the number of cells that express cFos in the medial amygdala and in the GrCL and MCL layers of the AOB (Kondo et al., 2003). Thus, genital stimulation can modify the activity of the OB, and this stimulation can be fundamental to increase the number of new cells that arrive to the AOB.

In the present study no changes in the number of new cells were observed in the group exposed to a sexually receptive female. This observation is at variance with reports showing that scent-rich environments and sexually-relevant odors induce an increase in the number of new cells in the OB (Mak et al., 2007; Larsen et al., 2008; Oboti et al., 2009). However, in those studies experimental animals were continuously exposed to the odors for at least two days, whereas in our experiment the males were exposed to the female odors for only 1 h. It has been proposed that short term exposure to odorants does not increase the survival of new cells, instead their survival requires the discrimination, association, and formation of olfactory memory (Alonso et al., 2006; Mouret et al., 2008; Lazarini and Lledo, 2011). It is also possible that acute or chronic exposure to sexually relevant odors might have a different effect on OB neurogenesis, but in the present experiment the induction of new cells by mating does not appear to be associated with female odors. Further support for this contention comes from a recent study where females allowed to pace the sexual interaction showed an increase in the number of new cells in the GrCL of the AOB, while females exposed to sexually experienced males did not show changes in the number of cells in the this layer (Corona et al., 2011b). Similar observations have been described in the hippocampus after sexual behavior in males. Subjects exposed to a sexually receptive female, with whom they could copulate for 14 days had more neurons in the dentate gyrus than those males exposed to non-receptive females (Leuner et al., 2010). In this case males were mated in a conditioned where they controlled the sexual interaction. Thus, pacing the sexual interaction induces genital stimulation and reward value that are quantitatively and qualitatively different from non paced mating. These differences can modulate the number of new cells.

No significant differences in the number of new cells were found in the MOB and in the GlCL and MCL of the AOB. It has been demonstrated in mice that, while the number of new cells that reach the GrCL of the AOB is maximum 7 days after BrdU administration, the new granular cells in the MOB reach their maximum levels 15 days after the administration of the DNA synthesis marker (Oboti et al., 2009). Also, the majority of the new cells that reach the OB differentiate into granular cells and only a rare proportion migrate into the glomerular layer reaching their maximum level 1 month after BrdU administration (Petreanu and Alvarez-Buylla, 2002; Winner et al., 2002). Future studies are been planned to evaluate proliferation in the SVZ and RMS 2 days after mating and neurogenesis in the OB 45 days after sexual stimulation and determine if some cells integrate in the MOB or other layers of the AOB.

### Conflict of interest statement

The authors declare that the research was conducted in the absence of any commercial or financial relationships that could be construed as a potential conflict of interest.
